# Mortality-based definition of renal hyperfiltration in middle-aged men: a 35-year cohort from Finland

**DOI:** 10.1007/s11255-021-03048-6

**Published:** 2021-11-03

**Authors:** Mounir Ould Setti, Salah Eddine Oussama Kacimi, Leo Niskanen, Tomi-Pekka Tuomainen

**Affiliations:** 1 Institute of Public Health and Clinical Nutrition, University of Eastern Finland, Kuopio, Finland; 2 Global Database Studies, IQVIA, Espoo, Finland; 3 Faculty of Medicine, University of Tlemcen, Tlemcen, Algeria; 4 Department of Internal Medicine, Päijät-Häme Central Hospital, Lahti, Finland

**Keywords:** Mortality, Glomerular filtration rate, Renal hyperfiltration

## Abstract

**Background:**

While the impact of low glomerular filtration rate (eGFR) on various outcomes has been extensively studied, the other adverse occurrence, renal hyperfiltration (RHF), remains understudied, poorly defined, and, therefore, its impact on mortality unestablished.

**Methods:**

Using a population-based subcohort from the Kuopio Ischaemic Disease Risk Factor Study restricted to non-diabetic Finnish men aged 54 or 55 years, we followed up *n* = 1179 study participants for up to 35 years. We evaluated the hazard of all-cause mortality associated to RHF at different cutoff points defining eGFR. Based on models’ accuracy we suggested an optimal eGFR cutoff point for the definition of RHF. We divided the RHF category to three subgroups and evaluated them in terms of baseline characteristics and mortality hazard.

**Results:**

The eGFR value of 97 mL/min/1.73 m^2^ corresponded to the models with the highest accuracy. Overall RHF associated with an increased risk of mortality (hazard ratio [HR] 1.42; 95% confidence interval [CI] 1.21 to 1.67). Moderate RHF associated with a decreased HR of mortality when compared to mild (0.64; 95% CI 0.46 to 0.9) or to extreme RHF (0.61; 95% CI 0.43 to 0.85), suggesting a rather U-shaped relationship between RHF’s eGFR values and mortality hazard.

**Conclusion:**

The burden of increased eGFR within what is still considered normal eGFR category was highly underestimated. RHF’s eGFR values had a U-shaped association with the risk of overall mortality. A more uniform consensual definition of RHF is needed, as higher to normal eGFR values that are not without consequences.

**Supplementary Information:**

The online version contains supplementary material available at 10.1007/s11255-021-03048-6.

## Introduction

Glomerular filtration rate (GFR) stands as a key marker of renal function and an important predictor of morbimortality. Lower than normal GFR levels are known to be associated with cardiovascular disease and mortality, partly through kidney damage in chronic kidney disease (CKD) [1]. Higher than normal GFR, also termed renal hyperfiltration (RHF), has also been associated with CKD [2] and some conditions such as hypertension and diabetes mellitus [3], but its independent association with the overall risk of mortality has only been recently established [4, 5]. While the exact mechanism associating RHF to mortality is not yet determined, RHF has been proposed as potentially associated with low-grade systemic inflammation, oxidative stress, and thus, cardiorenal metabolic risk [6]. At any rate, higher than normal GFR association with mortality is not without consequence. Nevertheless, a definition distinguishing RHF from normal GFR is strikingly absent from the literature.

GFR estimation is subject to inaccuracy as it is, in most studies, indirectly estimated from serum creatinine concentration which can be influenced by diet and muscle mass. GFR varies also according to gender and ethnicity, and declines naturally with aging [7]. While the Chronic Kidney Disease Epidemiology Collaboration (CKD-EPI) method is emerging as the standard for clinical estimation of GFR, studies use a variety of methods to estimate GFR based on creatinine clearance [8]. Consequently, there is no consensus on the cutoff point of estimated GFR (eGFR) to define RHF [9, 10]. The threshold of the high eGFR category in 151 studies examined by Cachat et al. [9] ranged from 90.7 to 175 ml/min per 1.73 m^2^, with the majority of cutoff values varying between 115 and 150 ml/min per 1.73 m^2^. Similarly, in a systematic review by Kanbay et al. [11], among the 19 identified studies investigating the relation between high eGFR and mortality, 3 studies defined RHF, or the highest eGFR category, as the value of eGFR > 95th percentile after adjustment for age and sex, while most of the rest used an eGFR threshold varying between 90 and 125 ml/min per 1.73 m^2^.

In the absence of a definition for RHF, the burden of the disease could be underestimated as patients with a pathological eGFR could be classified as normal [12]. In addition, the effect of the variation of eGFR in RHF remained understudied [13]. Different levels of eGFR within the RHF category might have distinct effects on health and could indicate subtypes of RHF with possible contrasting physiopathological and clinical characteristics. The purpose of this study was (i) to determine an optimal definition of RHF, in a population restricted to non-diabetic Finnish men of the same age (54 to 55 years), by investigating the long-term hazard of all-cause mortality associated to RHF defined using different cutoff values of GFR, and (ii) to evaluate the risk of mortality within subgroups of RHF.

## Methods

### Data source and variable measurement

Our study is based on a subcohort of 1592 Finnish men from the population-based Kuopio Ischaemic Heart Disease Risk Factor Study who were aged 54 or 55 years during their baseline examination between 1984 and 1989. After excluding subjects with diabetes at baseline (*n* = 107), subjects who reported

abstinence from alcohol consumption during the year preceding study enrollment (*n* = 239), outliers (*n* = 8), and subjects with missing values, we settled for *n* = 1179 men who were then followed up for a median of 27 years and a maximum of 35 years in which their status of health was annually assessed. There was no loss to follow-up among the study participants.

At baseline, the participants were physically examined by a physician, and interviewed and blood-sampled by a study nurse [14]. Based on the CKD-EPI equation and using baseline serum creatinine adjusted according to the Jaffe method, we computed the eGFR, our main exposure of interest, expressed as units of mL/min/1.73 m^2^ [15, 16]. The eGFR cutoff to define RHF was a function of a sensitivity analysis. We set 60 mL/min/1.73 m^2^ as the cutoff value of eGFR to separate the normal from the low eGFR category. A sensitivity analysis also considered 77 mL/min/1.73 m^2^ as an eGFR cutoff value of separating the normal from the low eGFR category as recommended for our population’s age category by HUS, the largest health care provider in Finland [17]. The cause-of-death registry provided information on our outcome of interest: all-cause mortality.

### Data analysis

By means of R version 4.0.3 (https://www.R-project.org) we built crude and adjusted (for baseline body mass index (BMI) [18], smoking [current, previous, never smoker] [19], BMI-smoking interaction [20], alcohol consumption [gram per week] [21], vitamin D level[22] [deficiency defined as values below the 10th percentile corresponding to 23.08 nmol/L], and hypertension [defined as a positive history of hypertension, anti-hypertensive medication, or a mean blood pressure ≥ 140/90 mmHg]) Cox proportional hazard models evaluating the risk of mortality associated to RHF in comparison to the normal eGFR category. We defined the period at risk as the time from enrollment to death or to 31 December 2018.

We performed a sensitivity analysis to define RHF based on different cutoff points of eGFR separating the normal eGFR category from the RHF category with eGFR cutoff points ranging from and including 90 [11] to 110.54 mL/min/1.73 m^2^ (the 99.5 percentile) with a model each 0.1 mL/min/1.73 m^2^. We constructed receiver operating characteristic curves for each of the models. Based primarily on their related area under the curve (AUC) and secondarily on the models’ R-squared values, we identified high accuracy models and suggested an optimal eGFR cutoff point for the definition of RHF.

In addition, we divided the RHF category (after defining an optimal eGFR cutoff point) to three equivalent subgroups (cuts at 33.33 and 66.66 percentiles among men with RHF): mild RHF, moderate RHF, and extreme RHF. Using crude and adjusted Cox proportional hazard models, we assessed the hazard of mortality associated to each of the RHF subgroups in comparison to (i) the normal eGFR category, (ii) mild RHF, and (iii) moderate RHF. Finally, we compared the participants’ characteristics across all categories of eGFR and across RHF subgroups using Chi-square and Kruskal–Wallis tests.

## Results

**Fig. 1:**
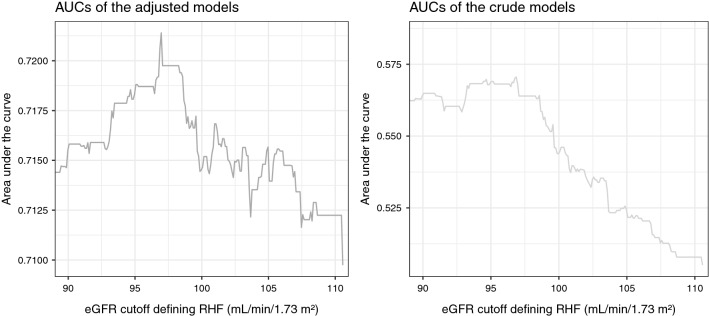
Area under the curve (AUC) for the discriminatory accuracy of the adjusted and the crude Cox regression models by changes of the estimated glomerular filtration rate’s (eGFR) cutoff point defining renal hyperfiltration (RHF). The adjusted models were adjusted for body mass index, smoking, the interaction between body mass index and smoking, alcohol consumption, hypertension, and vitamin D deficiency

**Fig. 2:**
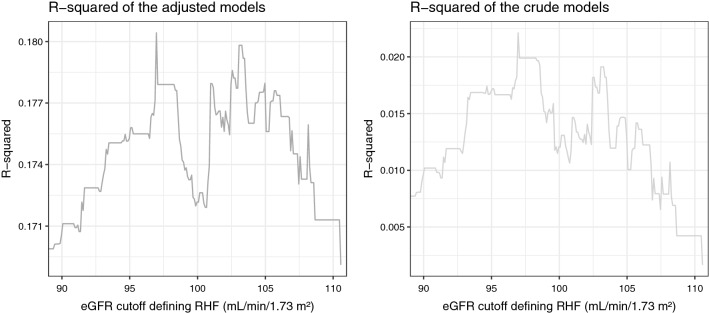
R-squared for the goodness-of-fit of the adjusted and the crude Cox regression models by changes of the estimated glomerular filtration rate’s (eGFR) cutoff point defining renal hyperfiltration (RHF). The adjusted models were adjusted for body mass index, smoking, the interaction between body mass index and smoking, alcohol consumption, hypertension, and vitamin D deficiency

We recorded 826 deaths during follow-up. Figure 1 illustrates changes of the AUC of adjusted and crude models by changes of the cutoff points defining RHF in these models. The eGFR value of 97 mL/min/1.73 m^2^ had the highest AUC in both adjusted (0.721; 95% confidence interval [CI] 0.693 to 0.750) and crude models (0.568; 95% CI 0.543 to 0.593). This result conformed with the R-squared plots of the crude and adjusted models (Fig. 2) and with the sensitivity analysis which set 77 mL/min/1.73 m^2^ as the cutoff eGFR between the low and the normal eGFR categories. Therefore, we considered 97 mL/min/1.73 m^2^ (77th percentile) as the optimal cutoff point between the normal eGFR and the RHF categories. Furthermore, the R-squared plots showed a second peak, slightly lower than the first, at around 103 mL/min/1.73 m^2^ (93rd percentile) with a corresponding AUC of 0.716 (CI, 0.687 to 0.744), hinting that more than one RHF cutoff point could be relevant to mortality hazard estimation. Overall RHF associated with an increased risk of mortality (hazard ratio [HR] 1.42; 95% CI 1.21 to 1.67).

**Fig. 3:**
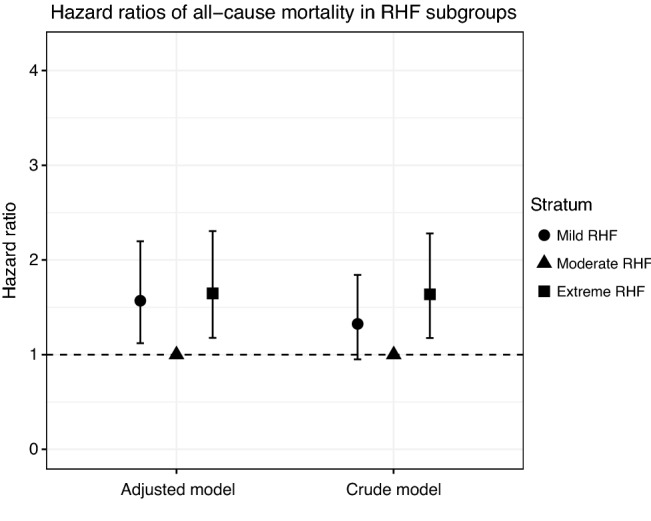
Crude and adjusted hazard ratios (HRs) with 95% confidence intervals (CI) for all-cause mortality in renal hyperfiltration’s (RHF) subgroups. Ref, reference category. The adjusted models were adjusted for body mass index, smoking, the interaction between body mass index and smoking, alcohol consumption, hypertension, and vitamin D deficiency

**Fig. 4:**
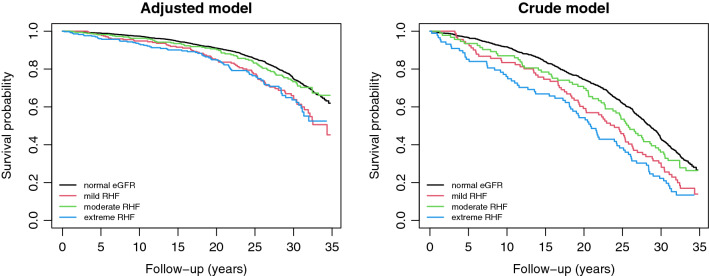
Adjusted and crude survival curves for all-cause mortality within renal hyperfiltration’s (RHF) subgroups. The adjusted models were adjusted for body mass index, smoking, the interaction between body mass index and smoking, alcohol consumption, hypertension, and vitamin D deficiency

We found a relation of direct proportion between the RHF-associated hazard of mortality and the eGFR threshold of RHF after an initial steady phase, suggesting a possible biological gradient between high eGFR values and the long-term risk of mortality (supplementary material 1). To investigate this, we divided the RHF category (≥ 97 mL/min/1.73 m^2^) into three equivalent subgroups (cuts at 33.33 and 66.66 percentiles among men with RHF): mild (97 to 99.96 mL/min/1.73 m^2^), moderate (99.96 to 102.44 mL/min/1.73 m^2^), and extreme RHF (≥ 102.44 mL/min/1.73 m^2^); and compared them in term of baseline characteristics and hazard of overall mortality. The study participants did not differ in status of mortality, BMI, smoking status, or alcohol consumption across RHF subgroups although they differed across the ensemble of eGFR categories in respect to these characteristics (Table 1). Surprisingly, moderate RHF associated with a decreased hazard of mortality when compared to mild (HR, 0.64; 95% CI 0.46 to 0.89) or to extreme RHF (HR, 0.61; 95% CI 0.43 to 0.85), suggesting a rather U-shaped relationship between RHF’s eGFR values and the hazard of all-cause mortality (Figs. 3 and 4). The HRs of all-cause mortality are reported in supplementary material 2.

**Table 1:** Baseline characteristics and follow-up differences by estimated glomerular filtration rate (eGFR)

	Total	Low eGFR (< 60)^a^	Normal eGFR (60 to 97)^a^	Mild RHF (97 to 99.96)^a, b^	Moderate RHF (99.96 to 102.44)^a, b^	Extreme RHF (≥ 102.44)^a, b^	Statistical test (all subgroups, *n* = 1187)^c^	Statistical test (RHF subgroups, *n* = 269)^c, d^
*n*	1187	22	896	90	92	87		
Deaths (column %)	826 (69.59)	14 (63.64)	597 (66.63)	74 (82.22)	67 (72.83)	74 (85.06)	Chi-square: df = 4, X2 = 21.16, *p* < 0.001	Chi-square: df = 2, X2 = 4.61, *p* = 0.1
Age in years	54.42 [54.33, 54.50]	54.50 [54.33, 54.77]	54.42 [54.33, 54.50]	54.33 [54.17, 54.50]	54.33 [54.31, 54.42]	54.33 [54.33, 54.50]	Kruskal–Wallis: df = 4, H = 20.62, *p* < 0.001	Kruskal–Wallis: df = 2, H = 1.35, *p* = 0.51
BMI (column %)							Chi-square: df^e^ = NA, X2 = 27.08, *p* = 0.041	Chi-square: df = 8, X2 = 12.12, *p* = 0.146
≤ 25	373 (31.42)	6 (27.27)	266 (29.69)	26 (28.89)	37 (40.22)	38 (43.68)		
(25, 27.5]	365 (30.75)	9 (40.91)	276 (30.80)	27 (30.00)	25 (27.17)	28 (32.18)		
(27.5, 30]	253 (21.31)	3 (13.64)	209 (23.33)	20 (22.22)	12 (13.04)	9 (10.34)		
(30, 32.5]	126 (10.61)	4 (18.18)	95 (10.60)	11 (12.22)	12 (13.04)	4 (4.60)		
> 32.5	70 (5.90)	0 (0.00)	50 (5.58)	6 (6.67)	6 (6.52)	8 (9.20)		
Smoking status (column %)							Chi-square: df = 8, X2 = 32.22, *p* < 0.001	Chi-square: df = 4, X2 = 8.99, *p* = 0.061
Never smoker	348 (29.32)	9 (40.91)	284 (31.70)	25 (27.78)	19 (20.65)	11 (12.64)		
Previous smoker	430 (36.23)	9 (40.91)	329 (36.72)	33 (36.67)	31 (33.70)	28 (32.18)		
Current smoker	409 (34.46)	4 (18.18)	283 (31.58)	32 (35.56)	42 (45.65)	48 (55.17)		
Alcohol consumption in g/week	40.80 [11.80, 96.68]	18.62 [5.43, 66.03]	38.45 [11.93, 94.95]	45.40 [14.48, 104.81]	48.00 [10.00, 103.69]	51.00 [11.50, 108.15]	Kruskal–Wallis: df = 4, H = 4.76, *p* = 0.313	Kruskal–Wallis: df = 2, H = 0.51, *p* = 0.774
Hypertension (column %)	727 (61.25)	15 (68.18)	559 (62.39)	52 (57.78)	53 (57.61)	48 (55.17)	Chi-square: df = 4, X2 = 3.26, *p* = 0.515	Chi-square: df = 2, X2 = 0.15, *p* = 0.926
Vitamin D deficiency (column %)	119 (10.03)	1 (4.55)	80 (8.93)	7 (7.78)	13 (14.13)	18 (20.69)	Chi-square: df^e^ = NA, X2 = 15.12, p = 0.006	Chi-square: df = 2, X2 = 6.08, p = 0.048
eGFR in mL/min/1.73 m^2^	85.17 [76.98, 96.83]	56.65 [50.40, 58.65]	81.95 [75.22, 88.57]	99.13 [98.57, 99.60]	100.80 [100.74, 101.92]	104.88 [103.64, 107.24]	Kruskal–Wallis: df = 4, H = 674.40, *p* < 0.001	Kruskal–Wallis: df = 2, H = 238.47, < 0.001
Follow-up in years	27.42 [18.06, 32.03]	26.23 [9.17, 30.01]	28.54 [19.73, 32.48]	23.68 [14.97, 30.25]	25.66 [16.41, 32.41]	20.84 [10.33, 28.47]	Kruskal–Wallis: df = 4, H = 37.47, *p* < 0.001	Kruskal–Wallis: df = 2, H = 8.41, *p* = 0.015
Age of death in years	81.82 [72.44, 86.25]	80.61 [63.52, 84.70]	83.03 [74.05, 86.85]	77.97 [69.39, 84.61]	79.95 [70.87, 86.79]	75.17 [64.95, 82.96]	Kruskal–Wallis: df = 4, H = 38.07, *p* < 0.001	Kruskal–Wallis: df = 2, H = 8.27, *p* = 0.016

Numbers indicate median [interquartile range] unless otherwise indicated

*BMI* body mass index in kg/m^2^, *eGFR* estimated glomerular filtration rate, *RHF* renal hyperfiltration

^a^In mL/min/1.73 m^2^

^b^RHF subgroups were determined by cutoffs at 33.33 and 66.66 percentiles of men with RHF

^c^Kruskal–Wallis rank-sum test and Pearson’s chi-squared test were used for across groups comparisons

^d^Values concern RHF subgroups only: mild RHF, moderate RHF, and extreme RHF

^e^Because of the low expected frequencies in some cells, Monte-Carlo simulation (based on 5 × 10^5^ replicates) was used to estimate *p*-value

## Discussion

Our study’s results can be summarized into two findings. The first one is that the optimal eGFR cutoff point for the definition of RHF could be much lower than the 95th percentile, often used to define RHF. After evaluating the 35-year hazard of all-cause mortality associated to high eGFR at a range of thresholds in 1187 non-diabetic Finnish men of the same baseline age, we estimated that the eGFR value of 97 mL/min/1.73 m^2^ could serve as an optimal cutoff point for the definition of RHF in our study settings. This value represents the 77th percentile of eGFR in our study population, suggesting that usage of, for example, the 95th percentile of eGFR to define RHF [4, 23, 24] would underestimate the mortality burden attributed to RHF. In addition, this finding implies that the mortality risk attributed to low eGFR levels has also been mostly underestimated in the literature when compared to a reference category carrying its own part of mortality risk. The second finding of our study is the U-shaped association between RHF’s eGFR values and the hazard of overall mortality with an increased mortality risk at mild and extreme RHF subgroups. This finding suggests the existence of a multitude of subtypes of RHF possibly manifesting with different eGFR levels and leading to disparate prognoses.

RHF is a frequently observed phenomenon known to be favored by conditions such as diabetes, obesity, smoking, and hypertension [11]. RHF was long regarded as an early sign of CKD and diabetic nephropathy deterioration. While the physiopathology of RHF is unclear, especially beyond its association with diabetes, a growing body of research has recently established RHF as an independent predictor of mortality [4, 5]. Nonetheless, there is no consensual definition for RHF [11]. So far, RHF has been clinically defined using either percentile-based definitions or arbitrarily-set eGFR cutoffs. Cachat et al. noted in their systematic review that most of the studies of RHF had no control group in their choice of the eGFR cutoff point defining RHF, and to their surprise, similar eGFR thresholds were used across age-groups, even in pediatric studies, while it is established that eGFR varies considerably over time [9]. Without an outcome-based definition of RHF, the burden of the condition cannot be properly assessed, screened, or prevented, while as Donfrancesco et al. (2013) suggested, even small changes in eGFR could affect mortality risk [25]. Recently, Kim et al. (2020) [12] examined the effects of RHF, defined at different eGFR thresholds, on mortality in a population-based cohort. The authors found an increased hazard of mortality with RHF defined at eGFR cutoff values starting from 83.8 mL/min/1.73 m^2^ in middle-aged men. This threshold corresponded to the 28th percentile of eGFR in this population. The association between RHF and mortality was statistically significant with eGFR values greater or equal than the 65th percentile, suggesting that up to 35% of Korean middle-aged men might be at increased risk of mortality due to RHF.

With an analogous design, we reached a similar conclusion as Kim et al.’s, that RHF is, in general, underestimated. Kim et al.’s clinically meaningful RHF threshold was, however, much lower than the one in our findings. Ethnicity could be an explanation of this difference, but more likely, our smaller sample size did not suffice to detect a significant effect on mortality at lower RHF thresholds. When it comes to the association between eGFR and mortality within the RHF category, our findings also differed from Kim et al. who did not note a heterogeneity in the relation between subgroups of RHF and death. Kim et al.’s findings were similar to Cox et al. (2008) who found an increased mortality risk in the higher RHF category in comparison to the lower one after dividing the RHF category into 2 subgroups [13]. In our study, we suggested a 3-subgroup division of the RHF category and found no difference in mortality risk between the lowest RHF category and the highest, but rather a U-shaped relation between mortality and eGFR levels within the RHF category.

With no loss to follow-up, our study benefited of a long follow-up time and a reliable assessment of the covariates and the mortality outcome. We restricted for age, gender, ethnicity, and the absence of diabetes and adjusted for smoking, obesity, the interaction between smoking and obesity, alcohol consumption, hypertension, and vitamin D deficiency. We found an association between RHF and increased mortality risk in both the crude and the adjusted models. Also, the ideal cutoff point of RHF was similar in the crude and adjusted models and when a more conservative eGFR cutoff point between the low and the normal eGFR category was considered. Our study is limited by the imperfection of eGFR in reflecting the real filtration rate of the kidneys. Calibration of our results using Iohexol clearance could bring more validity and reproducibility to our study findings [26]. Additional adjustment for grip strength [27], measures of central obesity such as muscle mass and waist-hip ratio [18], and dietary protein intake [11] could be an improvement for our study. We did not consider albumin level in our analysis, but Group et al. (2009) showed that the association of RHF with mortality is independent of albumin levels [28]. Also, serum creatinine from which we computed eGFR was measured only once, while the National Kidney Foundation’s Kidney Disease Outcomes Quality Initiative guidelines recommend using a second serum creatinine measurement after an interval of at least 90 days for clinical diagnosis. Moreover, our results concern only non-diabetic middle-aged Finnish men. A consensual definition of RHF would require its assessment in a more diverse population. Our exploration of RHF subtypes could also be extended by considering further stratification of RHF in a larger sample size. Finally, the multiphasic character of eGFR trajectory in RHF [29] violates the proportionality of hazards assumed in our methods. Consideration of the direction, rate, and trend of eGFR changes, and near-future occurrence of diabetes and chronic kidney disease would be necessary to explain the variation of mortality risk by RHF subtypes.

## Conclusion

The burden of increased eGFR within what is still considered normal eGFR category was highly underestimated. This could imply that the impact of low eGFR on mortality has also been underestimated. RHF’s eGFR values had a U-shaped association with the risk of overall mortality. A consensual definition of RHF is needed as higher to normal eGFR values are not without consequences.

## Supplementary Information

Below is the link to the electronic supplementary material.

Supplementary file1 (TIFF 9492 KB) Hazard ratios (HR) and p-values for the association of the renal hyperfiltration category (RHF) with all-cause mortality in reference to the normal estimated glomerular filtration rate’s (eGFR) category by changes of the eGFR cutoff point defining the RHF category. The adjusted models were adjusted for body mass index, smoking, the interaction between body mass index and smoking, alcohol consumption, hypertension, and vitamin D deficiency.

Supplementary file2 (DOCX 29 KB)

## Data Availability

The University of Eastern Finland can be approached for requests of access to the KIHD dataset.
