# Supplementary figures and images for: Mortality-based definition of renal hyperfiltration in middle-aged men: a 35-year cohort from Finland
Source: Int Urol Nephrol. 2021 Nov 3;54(7):1673–80. doi: 10.1007/s11255-021-03048-6 (PMC9184436; doi:10.1007/s11255-021-03048-6)

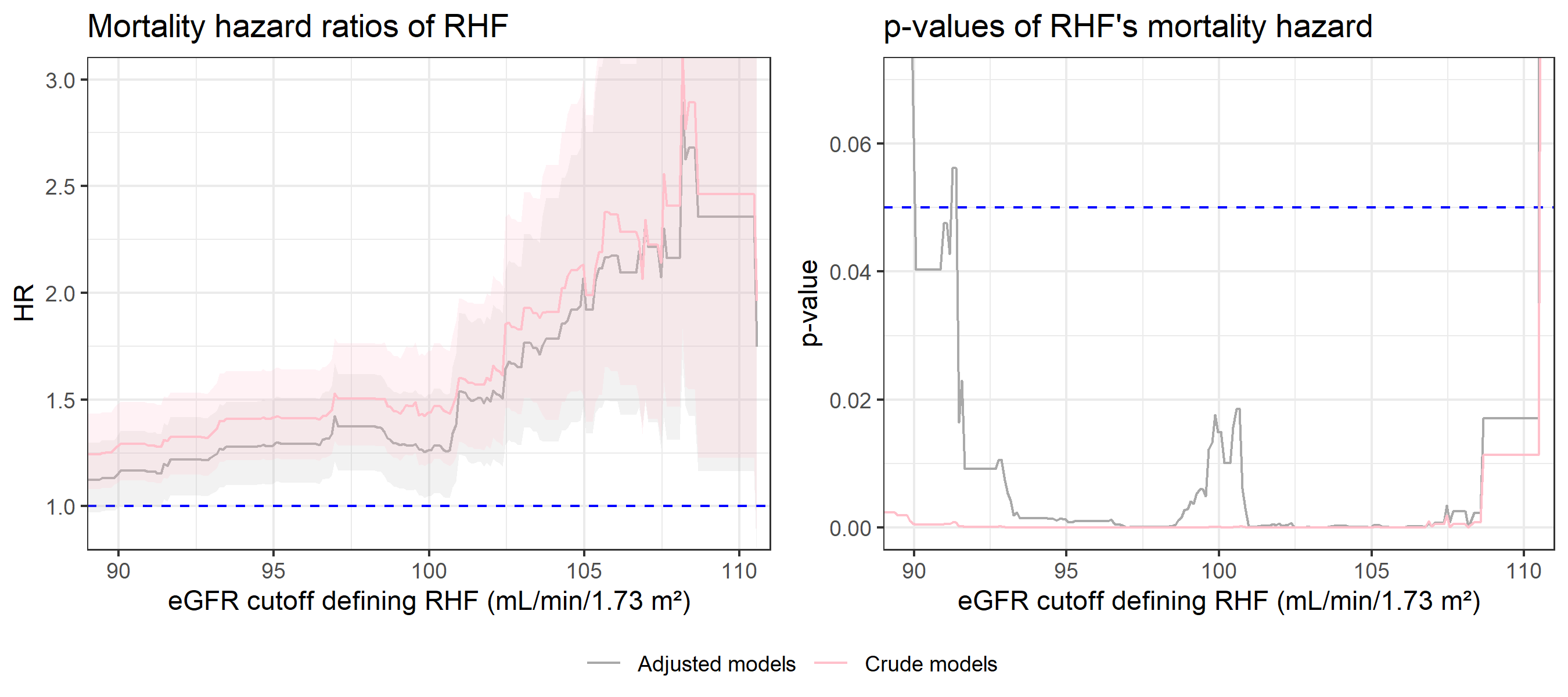

Supplement: Supplementary file 1 — Supplementary file1 (TIFF 9492 KB) Hazard ratios (HR) and p-values for the association of the renal hyperfiltration category (RHF) with all-cause mortality in reference to the normal estimated glomerular filtration rate’s (eGFR) category by changes of the eGFR cutoff point defining the RHF category. The adjusted models were adjusted for body mass index, smoking, the interaction between body mass index and smoking, alcohol consumption, hypertension, and vitamin D deficiency. [file 11255_2021_3048_MOESM1_ESM.tiff]
