# Supplementary material for: Mortality-based definition of renal hyperfiltration in middle-aged men: a 35-year cohort from Finland
Source: Int Urol Nephrol. 2021 Nov 3;54(7):1673–80. doi: 10.1007/s11255-021-03048-6 (PMC9184436; doi:10.1007/s11255-021-03048-6)
Supplement: Supplementary file 2 — Supplementary file2 (DOCX 29 KB) [file 11255_2021_3048_MOESM2_ESM.docx]

**Supplementary material 2.** Hazard ratios (HRs) of all-cause mortality in the study population (n=1187)

|  |  |  |  |
| --- | --- | --- | --- |
|  | **Number of events (n)** | **Crude HR (95% CI)** | **Adjusted HR (95% CI)** |
|  |  |  |  |
|  |  |  |  |
| Normal eGFR (ref.) | 597 (896) | 1 | 1 |
| Low eGFR | 14 (22) | 1.22 (0.72, 2.08) | 1.67 (0.98, 2.86) |
| Mild RHF | 74 (90) | 1.58 (1.24, 2.02) | 1.65 (1.29, 2.1) |
| Moderate RHF | 67 (92) | 1.2 (0.93, 1.54) | 1.05 (0.81, 1.36) |
| Extreme RHF | 74 (87) | 1.96 (1.54, 2.49) | 1.73 (1.35, 2.22) |
|  |  |  |  |
| Mild RHF (ref.) | 74 (90) | 1 | 1 |
| Low eGFR | 14 (22) | 0.77 (0.44, 1.37) | 1.02 (0.57, 1.81) |
| Normal eGFR | 597 (896) | 0.63 (0.5, 0.8) | 0.61 (0.48, 0.77) |
| Moderate RHF | 67 (92) | 0.76 (0.54, 1.05) | 0.64 (0.46, 0.89) |
| Extreme RHF | 74 (87) | 1.24 (0.9, 1.71) | 1.05 (0.76, 1.46) |
|  |  |  |  |
| Moderate RHF (ref.) | 67 (92) | 1 | 1 |
| Low eGFR | 14 (22) | 1.02 (0.58, 1.82) | 1.59 (0.88, 2.87) |
| Normal eGFR | 597 (896) | 0.84 (0.65, 1.08) | 0.95 (0.74, 1.23) |
| Mild RHF | 74 (90) | 1.32 (0.95, 1.84) | 1.57 (1.12, 2.2) |
| Extreme RHF | 74 (87) | 1.64 (1.18, 2.28) | 1.65 (1.18, 2.3) |
|  |  |  |  |
| Extreme RHF (ref.) | 74 (87) | 1 | 1 |
| Low eGFR | 14 (22) | 0.63 (0.35, 1.11) | 0.97 (0.54, 1.73) |
| Normal eGFR | 597 (896) | 0.51 (0.4, 0.65) | 0.58 (0.45, 0.74) |
| Mild RHF | 74 (90) | 0.81 (0.59, 1.12) | 0.95 (0.69, 1.32) |
| Moderate RHF | 67 (92) | 0.61 (0.44, 0.85) | 0.61 (0.43, 0.85) |
|  |  |  |  |
| BMI (kg/m2) ≤ 25 (ref.) | 230 (373) | 1 | 1 |
| (25, 27.5] | 253 (365) | 1.13 (0.95, 1.35) | 1.6 (1.07, 2.4) |
| (27.5, 30] | 190 (253) | 1.38 (1.14, 1.68) | 2.24 (1.47, 3.41) |
| (30, 32.5] | 96 (126) | 1.39 (1.09, 1.76) | 2.24 (1.33, 3.79) |
| > 32.5 | 57 (70) | 1.99 (1.49, 2.67) | 2.99 (1.52, 5.89) |
|  |  |  |  |
| Never smoker | 181 (348) | 1 | 1 |
| Previous smoker | 297 (430) | 1.59 (1.32, 1.91) | 1.75 (1.16, 2.63) |
| Current smoker | 348 (409) | 2.82 (2.35, 3.37) | 4.81 (3.35, 6.89) |
|  |  |  |  |
| Alcohol consumption in 100g/week | - | 1.24 (1.17, 1.32) | 1.14 (1.07, 1.22) |
|  |  |  |  |
| No hypertension | 292 (460) | 1 | 1 |
| Hypertension | 534 (727) | 1.31 (1.13, 1.51) | 1.26 (1.08, 1.46) |
|  |  |  |  |
| Normal vit. D level | 735 (1068) | 1 | 1 |
| Vit. D deficiency | 91 (119) | 1.2 (0.96, 1.49) | 1.12 (0.9, 1.39) |
|  |  |  |  |
| BMI (kg/m2) ≤ 25 : never smoker (ref.) | 40 (112) | 1 | 1 |
| (25, 27.5] : previous smoker | 80 (115) | 0.34 (0.2, 0.59) | 0.82 (0.48, 1.39) |
| (27.5, 30] : previous smoker | 77 (105) | 0.41 (0.24, 0.7) | 0.72 (0.42, 1.23) |
| (30, 32.5] : previous smoker | 54 (70) | 0.45 (0.26, 0.78) | 0.71 (0.37, 1.35) |
| > 32.5 : previous smoker | 30 (37) | 0.57 (0.31, 1.05) | 0.64 (0.29, 1.44) |
| (25, 27.5] : current smoker | 116 (140) | 0.54 (0.32, 0.91) | 0.47 (0.29, 0.75) |
| (27.5, 30] : current smoker | 62 (69) | 0.74 (0.43, 1.29) | 0.45 (0.27, 0.75) |
| (30, 32.5] : current smoker | 20 (24) | 0.64 (0.33, 1.23) | 0.37 (0.18, 0.75) |
| > 32.5 : current smoker | 16 (18) | - | 0.43 (0.18, 1.02) |
|  |  |  |  |
| BMI, body mass index; CI, confidence interval; eGFR, estimated glomerular filtration rate; ref., reference category; RHF, renal hyperfiltration; vit., vitamin. Crude HRs were unadjusted hazard ratios. Adjusted HRs were adjusted for body mass index, smoking, the interaction between body mass index and smoking, alcohol consumption, hypertension, and vitamin D deficiency. | | | |
